# Pre-diagnosis urine exosomal RNA (ExoDx EPI score) is associated with post-prostatectomy pathology outcome

**DOI:** 10.1007/s00345-022-03937-0

**Published:** 2022-01-27

**Authors:** Alexander Kretschmer, Ronald Tutrone, Jason Alter, Elena Berg, Christian Fischer, Sonia Kumar, Phillipp Torkler, Vasisht Tadigotla, Michael Donovan, Grannum Sant, Johan Skog, Mikkel Noerholm

**Affiliations:** 1 Deparment of Urology, LMU-Klinikum der Universität München, Munich, Germany; 2 United Urology Group, Towson, MD USA; 3 Exosome Diagnostics, 266 2nd Ave #200, Waltham, MA 02451 USA; 4 Department of Pathology, The University of Miami, Miami, FL USA

**Keywords:** Exosomes, PSA-gray-zone, Urine-biomarker, Radical-prostatectomy, Upgrading, Prostate cancer

## Abstract

**Purpose:**

ExoDx Prostate *IntelliScore* (EPI) is a non-invasive urine exosome RNA-based test for risk assessment of high-grade prostate cancer. We evaluated the association of pre-biopsy test results with post-radical prostatectomy (RP) outcomes to understand the potential utility of EPI to inform invasive treatment vs active surveillance (AS) decisions.

**Methods:**

Urine samples were collected from 2066 men scheduled for initial biopsy with PSA between 2 and 10 ng/mL, no history of prostate cancer, and ≥ 50 years across multiple clinical studies. 310 men proceeded to RP, of which 111 patients had Gleason group grade 1 (GG1) at biopsy and would have been potential candidates for AS. We compared pre-biopsy urine scores with ERSPC and PCPT multivariate risk calculator scores for men with GG1 at biopsy to post-RP pathology.

**Results:**

Urine EPI scores were significantly lower in men with GG1 at biopsy than in men with > GG1 (*p* = 0.04), while there were no differences in multivariate risk scores used in standard clinical practice (*p* > 0.05). Further, EPI scores were significantly lower in men with GG1 at biopsy who remained GG1 post-RP compared to men upgraded to ≥ GG3 post-RP (*p* < 0.001). In contrast, none of the multiparametric risk calculators showed significant differences (*p* > 0.05). Men with GG1 at biopsy and EPI score < 15.6 had zero rate of upgrading to ≥ GG3 post-RP compared to 16.0% for EPI scores ≥ 15.6.

**Conclusions:**

The EPI urine biomarker outperformed the multivariate risk calculators in a homogenous risk group of pre-biopsy men. The EPI score was associated with low-risk pathology post-RP, with potential implications on informing AS decisions.

**Trial registration:**

NCT02702856, NCT03031418, NCT03235687, NCT04720599.

**Supplementary Information:**

The online version contains supplementary material available at 10.1007/s00345-022-03937-0.

## Introduction

Prostate cancer (PC) is a leading cause of cancer death among men in the United States, affecting more than 3.6 million men. It is estimated that 248,530 new cases will be diagnosed in 2021, and 34,130 will die from the disease [1]. Active surveillance (AS) is recommended for men diagnosed with low-risk PC [e.g. Gleason grade group 1 (GG1)], and the National Comprehensive Cancer Network (NCCN) guidelines suggest that also men with low-volume GG2 may be candidates for AS. Routine clinical decision-making in AS candidates includes several challenges such as biopsy sampling error, individual genetic variability, and the multifocal nature of the disease. Tumor heterogeneity represents an essential limitation for tissue-based genomic tests since multifocal tumors in the same prostate can have different histology and genomic diversity [2, 3]. Additionally, despite the recent advances in the use of magnetic resonance imaging (MRI) for patient risk assessment prior to biopsy, the role of MRI in guiding decisions for active surveillance has yet to be established [4]. We hypothesized that a liquid biomarker-based assay, which provides a more global assessment of the entire prostate gland than a tissue biopsy, will provide a more relevant risk assessment for men enrolled in or considered for AS [5].

The ExoDx Prostate *Intelliscore* (EPI) test, is a urine-based biomarker assay that relies on isolation and analysis of gene expression in urinary exosomes, and which does not require digital rectal examination prior to sample collection. Exosomes are small vesicles (typically 30–200 nm in diameter) released from cells and surrounded by a lipid bilayer membrane that protects the RNA cargo inside, making it possible to profile the molecular content of the tumor from a biofluid sample. Exosomes contain RNA, DNA, and proteins and are particularly useful for RNA expression profiling given their protected microanatomic environment, which preserves the RNA [6]. The EPI test has been validated in multiple prospective clinical studies to assess the risk of high-grade PC (≥ GG2) in men with an equivocal PSA (2–10 ng/mL), prior to initial or repeat prostate biopsy [7–11]. In this setting, EPI demonstrated both a high negative predictive value (NPV) of 91.3% (≥ GG2) and 97% (≥ GG3) and a sensitivity of 92% at a cut point of 15.6 [7, 8].

Since the potential for upstaging and upgrading represents a major concern associated with the use of AS, the current analysis investigates the possible utility of the EPI test to inform decision-making in men considered for AS. We evaluate the association of the EPI biomarker score in urine samples collected pre-biopsy diagnosis with pathology outcome in men who subsequently proceeded with RP, and we compare EPI test performance with established multiparametric risk calculators that include factors used in clinical practice.

## Methods and materials

### Study population

Urine samples were collected from men scheduled for initial biopsy in multiple clinical studies (NCT02702856, NCT03031418, NCT03235687 and NCT04720599) from 2014 through 2020 as previously described [7–9, 12]. All urine samples were collected without prior digital rectal examination or prostate massage. Among the men enrolled in these studies who proceeded to RP, a subset with GG1 biopsy pathology (*N* = 111) would have been potential candidates for AS. Inclusion criteria at the time of enrollment and testing included no history of PC or previous biopsy, ≥ 50 years, and PSA between 2 and 10 ng/mL. Patients with a history of invasive treatment, current urinary tract infections or 5-alpha-reductase inhibitors due to benign prostatic hyperplasia within six months before testing were excluded.

### Sample collection and processing

First catch urine samples (20–50 mL) were stored at 4 °C for up to 14 days before shipment to an Exosome Diagnostics central laboratory (either CLIA Lab, Waltham, MA, US or ISO15189 Lab, Martinsried, Germany). Samples were filtered (0.8 µm) and immediately processed or stored at − 80 °C until further processing. For each sample, exosomal RNA was extracted, and the RNA expression levels of PCA3 (prostate cancer antigen 3), ERG (V-ets erythroblastosis virus E26 oncogene homologs), and SPDEF (SAM Pointed Domain Containing ETS Transcription Factor) were determined as previously described [7, 10].

### Statistical analysis

The EPI performance was compared to existing clinical features (PSA, age, race, and family history) and multivariate risk calculators from the Prostate Cancer Prevention Trial (PCPT) and European Randomized Prostate Cancer Study of Screening for Prostate Cancer (ERSPC). All statistical analyses and plots were generated using R version 4.0.3. Statistical differences in the clinical and demographic factors of categorical variables were estimated using Pearson’s chi-squared test. Continuous variables were tested for normality using the Shapiro–Wilk test. Parametric distributions of two variables were compared using a Welch two-sample *t* test, and more than two normally distributed variables were compared using one-way ANOVA. Non-parametric distributions of more than two variables were compared using a Kruskal–Wallis rank sum test followed by Dunn’s test for multiple pairwise comparisons using Benjamini–Hochberg adjustment for *p* values < 0.05.

## Results

### Patient characteristics and tissue analysis

Of the pre-diagnosis urine samples collected from 2066 men scheduled for an initial biopsy [7–9, 12], 310 were from men who subsequently underwent RP. In these 310 men, the median age at diagnosis was 62 years, while the median PSA was 5.4 ng/mL. Among the patients with GG1 (*N* = 111) and > GG1 (*N* = 199) at biopsy, there were no significant differences in PSA value (median PSA 5.3 [inter-quartile-range (IQR) 4.3–6.5] vs 5.4 [IQR 4.3–7] ng/mL; *p* = 0.46), African American ethnicity (7.2% vs 10.5%; *p* = 0.57), or family history of PC (32% vs. 24%; *p* = 0.29). Men with higher post-RP pathology (> GG1) were found to be significantly older (60 [IQR 59–62] vs 64 [IQR 63–65] years; *p* < 0.0001). Furthermore, EPI scores from patients in the biopsy > GG1 group (median 40: 95% CI 39–44) were found to be significantly higher than for patients in the GG1 group (median 32: 95% CI 33–40, *p* < 0.05. Detailed patient characteristics are described in Table S1.

### Post-RP analysis

Of the 111 patients with GG1 on biopsy, 62 were > GG1 in the post-RP pathology, corresponding to an overall probability of upgrading from biopsy GG1 of 56% across the cohort. Amongst the 62 patients, 47 (42%) and 15 (14%) were upgraded to GG2 and ≥ GG3, respectively. Details of all up- and down-gradings are described in Table S2.

**Fig. 1:**
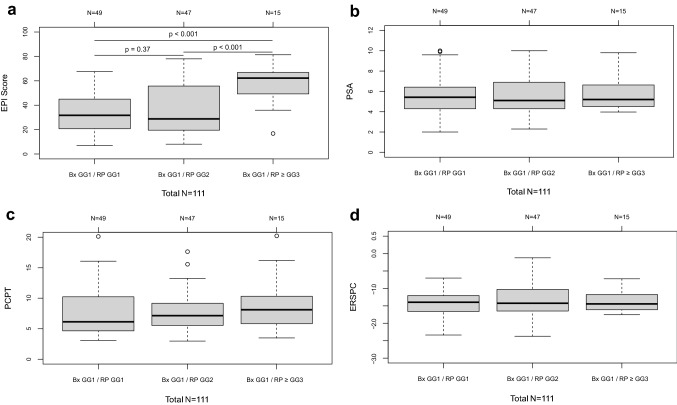
EPI/PSA/PCPT/ERSPC score vs RP upgrading. Distribution scores of different parameter for patients with GG1 at biopsy who (1) remained GG1 post- RP, (2) were upgraded to GG2 post-RP, and (3) were upgraded to > GG3 post-RP for **A** EPI, ExoDx™ Prostate (IntelliScore) (*p* values for group comparisons shown in the figure); **B** PSA levels (Kruskal–Wallis rank sum test, *p* = 0.95 for comparison of all three groups); **C** PCPT risk calculator (Kruskal–Wallis rank sum test, *p* = 0.29); and **D** ERSPC risk calculator (one-way ANOVA, *p* = 0.76)

Of the 49 men (44%) with GG1 at biopsy who remained GG1 post-RP, EPI test scores were significantly lower (median score of 31.7 [IQR 20.7–45.0]) compared to men who were subsequently upgraded to ≥ GG3 post-RP (62.2 [IQR 49.3–66.8], *p* < 0.0001) (Table 1 and Fig. 1A). EPI scores in men who were upgraded to GG2 (28.8 [IQR 19.5–55.7]) failed to reach significance (*p* = 0.37) (Fig. 1A). In contrast, neither PSA levels (Fig. 1B) nor the existing risk assessment tools including the PCPT (Fig. 1C) or ERSPC risk calculators (Fig. 1D), demonstrated any significant differences between these groups.

**Table 1:** Demographics and clinical characteristics for all patients with Gleason Grade Group (GG) 1 on biopsy and subgroups based on Radical Prostatectomy outcome

Radical-prostatectomy group	Biopsy GG1			
	All	Confirmed GG1	Upgrade to GG2	Upgrade to > GG2
Number of patients, *N*	111	49	47	15
Age (at diagnosis), median (IQR)	60.0 (57.0–65.0)	58.0 (56.0–63.0)	60.0 (56.0–65.0)	60.0 (58.0–69.5)
PSA (ng/mL), median (IQR)	5.35 (4.3–6.5)	5.4 (4.3–6.4)	5.1 (4.3–6.9)	5.2 (4.5–6.6)
African American, *n* (%)	8 (7.2%)	4 (8.2%)	3 (6.4%)	1 (6.7%)
Positive family history, *n* (%)	35 (31.5%)	19 (38.8%)	12 (25.5%)	4 (26.7%)
EPI Score, median (IQR)	32.4 (20.6–55.2)	31.7 (20.7–45.0)	28.8 (19.5–55.7)	62.2 (49.3–66.8)
Radical-prostatectomy outcome, *n* (%)				
GG1	49 (44.1%)	49 (100.0%)	–	–
GG2	47 (42.3%)	–	47 (100.0%)	–
GG3	12 (10.8%)	–	–	12 (80.0%)
GG4	1 (0.9%)	–	–	1 (6.7%)
GG5	2 (1.8%)	–	–	2 (13.3%)

**Fig. 2:**
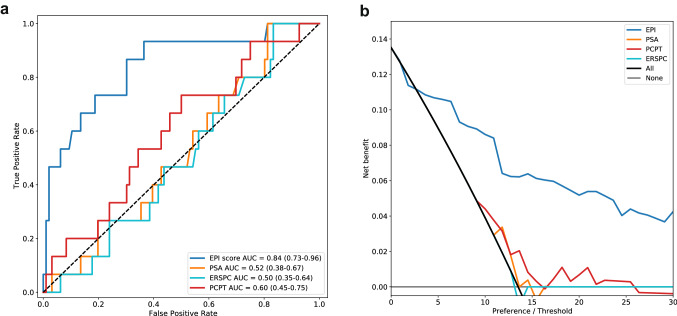
Receiver operator curves (ROC) (**A**) and decision curve/net benefit analysis (**B**) of EPI vs PSA, PCPT and ERSPC for prediction of RP GG > 2 after Bx GG1

Using prediction of GG > 2 at RP as a surrogate for patients being suitable candidates for AS, in a rank-based analysis, comparing the EPI test to existing alternatives, the EPI score had an area under the receiver-operator curve (AUC) of 0.84, which was significantly higher than both PSA (0.52, *p* = 0.00114), PCPT (0.60, *p* = 0.1397) and ERSPC (0.50, *p* = 0.00038), see Fig. 2A. Further, in a decision curve analysis [24, 25] (Fig. 2B) we found EPI-CE to provide a higher Net Benefit than any of the alternatives across a wide range of risk acceptance levels compared to PSA and the two multiparametric risk calculators. A similar analysis of predicting upgrading to any grade (≥ GG2) in patients with Bx GG1, did not show significant separation by any of the investigated methods (Supplemental Fig. 2).

**Fig. 3:**
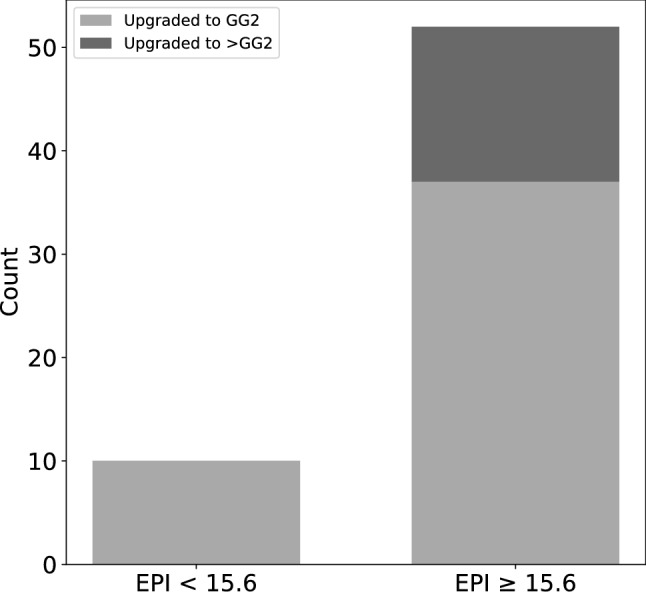
Pre-biopsy EPI score correlation with post-RP pathology by the previously established 15.6 EPI score cut point. No GG1 cases (0%) with an EPI score < 15.6 were upgraded to ≥ GG3 post-RP, compared to 16.0% upgraded to ≥ GG3 when EPI scores were ≥ 15.6 cut-point (*p* < 0.001). Fifty-eight percent of GG1 cases with an EPI score < 15.6 (10/17) were upgraded to GG2 post-RP compared to 55% (52/94) when EPI scores were ≥ 15.6 cut-point (*p* = 0.95)

The probability of upgrading from GG1 to post-RP ≥ GG3 was 13.5% (15/111) across the cohort. Men with GG1 at biopsy and an EPI score < 15.6 had a rate of zero (0 out of 17) for upgrading to ≥ GG3 post-RP compared to 16% (15 out of 94) when EPI scores were equal to or above the 15.6 threshold (*p* < 0.001) (Fig. 3). This equates to EPI having a Sensitivity and a Negative Predictive Value (NPV) of 100% for predicting ≥ GG3 pathology in patient with GG1 at biopsy in this cohort. Additional diagnostic parameters are listed in Table 2. However, this association was not observed in men with GG1 biopsy pathology who were subsequently upgraded to GG2 (Supplemental Fig. 1, Supplemental Table S3). Men with scores below or above the cut-point demonstrated similar rates of pathology upgrade [59% (10/17) with EPI < 15.6 vs. 55% (52/94) with EPI ≥ 15.6, (*p* = 0.95)]. Of note, all men in the cohort were initially diagnosed as GG1 cancer on biopsy (where EPI ≥ 15.6 would be a false positive), but 52 of these men were upgraded to GG2 or higher (true positive) upon RP vs only 10 men below the cut-point.

**Table 2:** Alternative EPI cut-points for use in the Bx-GG1 population, for predicting ≥ GG3 pathology at RP

Cut point	10	15.6	20	30	40	50
*N*	111	111	111	111	111	111
Sensitivity	100% (78–nan)	100% (78.2–nan)	93% (68–100)	93% (68–100)	87% (60–98)	73% (45–92)
Specificity	4% (1–10)	18% (11–27)	24% (16–34)	53% (43–63)	68% (57–77)	78% (69–86)
NPV	100% (40–nan)	100% (81–nan)	96% (79–100)	98% (90–100)	97% (90–100)	95% (88–99)
PPV	14% (8–22)	16% (9–25)	16% (9–26)	24% (14–37)	30% (17–45)	34% (19–53)
Diagnostic odds ratio	inf	inf	4.4	15.9	13.6	9.8
Samples below cut point	4% (1–9)	15% (9–23)	22% (14%–30%)	47% (37–57)	60% (51–70)	71% (62–79)

However, the 15.6 cut point was not intended for use in patients with confirmed GG1, but rather developed and validated for use of EPI in a pre-biopsy setting [7, 8, 10]. To explore potential use of a different cut-point for EPI in the Bx-GG1 patient population, we performed additional analysis using cut-points from 10 through 50 as listed in Table 2. For example, the EPI test maintains Sensitivity 93% and NPV 98% at a cut-point of 30, with 47% of patients thus having a score below the cut-point and potentially postponing the RP and considering them for AS.

### Discussion

Biopsy sampling error and prostate cancer multifocality causes many tumors to be assessed as low-risk at biopsy only to be upgraded post-RP because the identified tumor was not sampled adequately. In this study, more than half (56%) of the patients with a GG1 biopsy were upgraded post-RP. This is consistent with other studies on upgrading [12] and represents a challenge in the interpretation of biopsy results and deciding which patients need to proceed to RP and ultimately leading to some patient having RP, who would have been better served with active surveillance.

Patients benefit from a layered risk assessment approach, including integrating clinical features and the use of prognostic gene expression tools to profile tumors and predict progression [14]. However, tissue-based gene expression is dependent on the tissue obtained and identified with biopsy [15–17] and suffer from the same limitations of sampling, multifocality and tumor heterogeneity as the biopsy itself. One study evaluated the genomic variability of three commercial tissue assays for men with PC and observed genomic diversity between intra-prostate tumors as well as discordant results between different tissue-based tests, suggesting that improved results may be obtained by averaging the values of two or more intra-prostate tumors [17].

A liquid biopsy test, which samples analytes from the entire prostate organ, could address both multifocality and biopsy sampling biases and provide a more relevant assessment of risk for men considering AS [5]. This study explored the applicability of an exosome-based, non-invasive urine test for men with low-risk disease considering AS. When EPI scores were evaluated for men with GG1 on biopsy, those men who were upgraded to ≥ GG3, had significantly higher scores compared to men that remained GG1 post-RP (Fig. 1A). In contrast, neither PSA nor any of the standard multiparametric risk calculators provided any discrimination between these groups (Fig. 1B–D). Further, in this cohort, zero cases were upgraded when the EPI scores were < 15.6, resulting in a high NPV (100%) for ruling out ≥ GG3. When scores were ≥ 15.6, 16% of GG1 cases were upgraded to ≥ GG3, similar to overall GG1 upgrading to GG3 across the cohort (14.3%).

Although higher EPI scores in this study were associated with biopsy GG1 upgrading to ≥ GG3 post-RP, this correlation was not statistically significant when GG1 was upgraded post-RP to GG2. One explanation for this could be the limited sample size, however, biology could also be an explanation where GG1 tumors and GG2 tumors are very similar and distinct from a cohort perspective from GG3 and higher, however this needs to be further evaluated. Alternatively, there could be a selection bias where men proceeding to RP are more likely to be at higher risk due to uncaptured clinical factors, however, these would have to be factors beyond those included in the multiparametric risk calculators, which showed no ability to discriminate even GG1 from ≥ GG3. Gleason 7 tumors are classified as either favorable-intermediate risk (GG2) or unfavorable-intermediate risk (GG3) based on dominant pattern and tumor volume, however, this bi-modal Gleason 7 risk assessment is debated [18, 19]. Increasing amounts of pattern 4 architecture are considered a significant predictor of adverse pathology and biochemical recurrence (BCR). Men with GG2 at biopsy have lower BCR-free survival but are not more likely to metastasize than those with Gleason GG1 at two years post-RP [20, 21]. In North America, patients with GG2 are frequently allocated to active surveillance following large AS-studies indicating that discrimination between GG1 and GG2 might not be crucial from a clinical point of view [22, 23]. Furthermore, men with GG3 and GG4 cancers may have similar pathological characteristics, while GG1 tumors may be biologically similar to GG2 tumors [21]. However, it is interesting to note, that although GG1 and GG2 patients are separated by pathology at RP, they are impossible to distinguish both by PSA, the multivariate risk calculators as well as by the EPI test, whereas the ≥ GG3 patients are clearly distinguishable by EPI, but by none of the other measures.

Although the present study provides promising new insights into the ability of a pre-diagnosis urine measurement to predict the outcome of RP and offer a potential benefit to AS candidates, this study has some limitations. Out of the 2066 patients enrolled, only 111 men with an initial GG1 biopsy who proceeded with RP were available to follow-up. In addition, it is unlikely that most of the patients in this study were on AS but may have proceeded to RP after the initial diagnostic biopsy, as we were unable to capture the circumstances and factors involved in the clinical decision-making process. Since the patients in this study all proceeded with definitive treatment (RP) and since we had insufficient data to precisely categorize patients as AS candidates according to recognized guidelines (e.g. NCCN [26] or EAU [27]), we used GG pathology on RP as an approximation. Future studies, needed to expand on this analysis should more closely align with AS guideline criteria and include a multivariable analysis to evaluate the independent performance of the EPI test and a comparison of the EPI urine-based test with a tissue-based test to evaluate the accuracy of results, overcoming tumor multifocality and heterogeneity. In addition, prior to further studies, the most appropriate cut-point for use in the new clinical situation should be defined. Since the previous 15.6 cut-point was chosen for a clinical situation where the majority of candidates are undiagnosed with PCa, it is to be expected that many men with confirmed GG1 will have EPI scores > 15.6. From the multiple cut-point analysis on the current cohort, it appears that a new cut-point around 30 would be appropriate for the Bx-GG1 confirmed population, but this will need to be validated in independent studies.

### Conclusion

The EPI test is a noninvasive, urine gene expression assay initially developed and intended to assist physicians and their patients in making more informed decisions about the need for prostate biopsy. In this analysis, using post-RP data and pre-diagnosis EPI scores from a homogenous risk group of men (PSA 2–10 ng/mL), lower EPI urine biomarker scores was the only parameter associated with high-risk pathology after RP in men with GG1 on biopsy, whereas multiparametric risk scores using existing standard clinical parameters were not. Taken together, a liquid biopsy approach may address the limitation of tumor heterogeneity not addressed by tissue-based molecular assessment tools and provide physicians and candidates for AS with more insight at diagnosis and improved clinical management of PC.

## Supplementary Information

Below is the link to the electronic supplementary material.

Supplementary file1 (DOCX 95 KB)

Supplementary file2 (XLSX 17 KB)

Supplementary file3 (XLSX 17 KB)

## Data Availability

Not applicable.
