# Supplementary material for: Pre-diagnosis urine exosomal RNA (ExoDx EPI score) is associated with post-prostatectomy pathology outcome
Source: World J Urol. 2022 Jan 27;40(4):983–9. doi: 10.1007/s00345-022-03937-0 (PMC8994717; doi:10.1007/s00345-022-03937-0)
Supplement: Supplementary file 1 — Supplementary file1 (DOCX 95 KB) [file 345_2022_3937_MOESM1_ESM.docx]

**Pre-diagnosis Urine Exosomal RNA (ExoDx EPI score) is associated with Post-Prostatectomy Pathology Outcome**

Alexander Kretschmer^1^, Ronald Tutrone^2^, Jason Alter^3*^, Elena Berg^1^, Christian Fischer^3^, Sonia Kumar^3^, Phillipp Torkler^3^, Vasisht Tadigotla^3^, Michael Donovan^4^, Grannum Sant^3^, Johan Skog^3^, Mikkel Noerholm^3^

1. LMU-Klinikum der Universität München, Dept of Urology, Munich, Germany
2. United Urology Group, Towson, Maryland
3. Exosome Diagnostics, Waltham, Massachusetts
4. The University of Miami, Dept of Pathology, Miami, Florida

**SUPPLEMENT**

**Supplemental Figure 1**

**
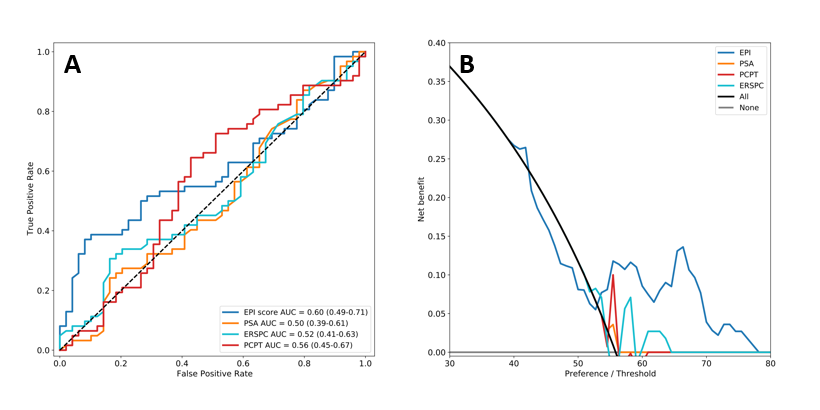
**

Supplemental Figure 1: Receiver Operator Curves (ROC) (A) and Decision Curve/Net Benefit Analysis (B) of EPI vs PSA, PCPT and ERSPC for prediction of RP ≥GG2 after Bx GG1.
